# Liver X Receptors (LXRs) in cancer-an Eagle’s view on molecular insights and therapeutic opportunities

**DOI:** 10.3389/fcell.2024.1386102

**Published:** 2024-03-14

**Authors:** Prasanna Srinivasan Ramalingam, Sujatha Elangovan, Janaki Ramaiah Mekala, Sivakumar Arumugam

**Affiliations:** ^1^ Protein Engineering Lab, School of Biosciences and Technology, Vellore Institute of Technology, Vellore, India; ^2^ Department of Biotechnology, Koneru Lakshmaiah Education Foundation (KLEF), Guntur, Andhra Pradesh, India

**Keywords:** liver X receptors, agonists, cancer, regulation, tumor microenvironment, precision medicine

## Abstract

Cancer has become a serious health burden that results in high incidence and mortality rates every year, mainly due to various molecular alterations inside the cell. Liver X receptors (LXRs) dysregulation is one among them that plays a vital role in cholesterol metabolism, lipid metabolism and inflammation and also plays a crucial role in various diseases such as obesity, metabolic dysfunction-associated fatty liver disease (MAFLD), cardiovascular diseases, Type 2 diabetes, osteoporosis, and cancer. Studies report that the activation of LXRs inhibits cancer growth by inhibiting cellular proliferation, inducing apoptosis and autophagy, regulating cholesterol metabolism, various signalling pathways such as Wnt, and PI3K/AKT, modulating the expression levels of cell-cycle regulators, and promoting antitumor immunity inside the tumor microenvironment. In this review, we have discussed the role, structure, and functions of LXRs and also summarized their ligands along with their mechanism of action. In addition, the role of LXRs in various cancers, tumor immunity and tumor microenvironment (TME) along with the importance of precision medicine in LXR-targeted therapies has been discussed to emphasize the LXRs as potent targets for the development of novel cancer therapeutics.

## 1 Role, structure, and functions of LXRs

Cancer has become a global health burden with constantly increasing incidence and mortality rates every year worldwide, and the quest for potent therapeutics development is still on in the era of precision medicine (Girisa et al., 2021; Ramalingam and Arumugam, 2023a; Ramalingam and Arumugam, 2023b; Ramalingam et al., 2023a; Ramalingam and Arumugam, 2023b). Alterations/modification in the function of genes/proteins triggers the oncogenic signalling pathways rather than the normal physiological conditions, and thus alterations induce cancer progression and tumorigenesis (Tarrado-Castellarnau et al., 2016). Nuclear receptors (NRs) are a class of proteins that act as eukaryotic transcription factors, regulate gene expression, mediate the transduction of signaling pathways, and are associated with various diseases such as cardiovascular diseases and cancer. To date, 48 NRs have been identified and their roles are being studied concerning their role in cellular development, homeostasis, and lipid and cholesterol metabolism (Sever and Glass, 2013; Mazaira et al., 2018; D’Arrigo et al., 2022). The 48 NRs are classified into 6 types, namely, Thyroid Hormone Receptor-like (NR1), Retinoid X Receptor-like (NR2), Estrogen Receptor-like (NR3), Nerve Growth Factor IB-like (NR4), Steroidogenic Factor-like (NR5), and Germ Cell Nuclear Factor-like (NR6) respectively (Tanaka et al., 2017; Tran et al., 2018). Moreover, based on their role and function they have been classified into 5 main types, namely, steroidogenesis (IA), reproduction and development (IB), CNS, circadian and basal metabolic functions (IC), bile acids and xenobiotic metabolism (IIA), lipid metabolism and energy homeostasis (IIB & IIC) respectively by Nuclear Receptor Signalling Atlas (NURSA) as shown in Table 1 (McKenna et al., 2009; Becnel et al., 2015; Parris, 2020; Königshofer et al., 2021; Peavey et al., 2022).

**TABLE 1:** Classification of nuclear receptors.

Function	Type	Nuclear receptors
Steroidogenesis	IA	DAX1 (dosage-sensitive sex reversal, adrenal hypoplasia critical region, on chromosome X, gene 1)
		SF-1 (Steroidogenic factor-1)
		FXRβ (Farnesoid X receptor-beta)
Reproduction and development	IB	AR (Androgen receptor)
		ERα (Estrogen receptors-alpha)
		ERβ (Estrogen receptor-beta)
		COUP-TFβ (COUP transcription factor-beta)
		RARα (Retinoic acid receptor-alpha)
		RARγ (Retinoic acid receptor-gamma)
		PR (progesterone receptor)
CNS, circadian and basal metabolic functions	IC	LXRβ (Liver X receptor-beta)
		RXRβ (Retinoid X receptor-beta)
		RXRγ (Retinoid X receptor-gamma)
		MR (Mineralocorticoid Receptor)
		TR4 (Testicular receptor 4)
		TLX (Homologue of the *drosophila* tailless gene)
		NOR-1 (Neuron-derived orphan receptor-1)
		NGF1B (Nerve growth factor 1B)
		RORα (Retinoid orphan nuclear receptor-alpha)
		RORβ (Retinoid orphan nuclear receptor-beta)
		Rev-Erbɑ (Rev-Erb-alpha)
		Rev-Erbβ (Rev-Erb-beta)
		RARβ (Retinoic acid receptor-beta)
		ERRβ (Estrogen-related receptor-beta)
		ERRγ (Estrogen-related receptor-gamma)
		NURR1 (Nuclear receptor related 1)
		COUP-TFα (COUP transcription factor-alpha)
		TRα (Thyroid hormone receptor-alpha)
Bile acids and xenobiotic metabolism	IIA	CAR (Constitutive androstane receptor)
		PXR (pregnane X receptor)
		FXRα (Farnesoid X receptor-alpha)
		HNF4α (Hepatocyte nuclear factor 4 alpha)
		HNF4γ (Hepatocyte nuclear factor 4-gamma)
		LRH-1 (Liver receptor homolog-1)
		SHP (Small heterodimer partner)
		RORγ (Retinoid orphan nuclear receptor-gamma)
		VDR (Vitamin D receptor)
Lipid metabolism and energy homeostasis	IIB	COUP-TFγ (COUP transcription factor-gamma)
		TRβ (Thyroid hormone receptor-beta)
		PPARα (Peroxisome proliferator-activated receptor-alpha)
		PPARδ (Peroxisome proliferator-activated receptor-delta)
		RXRα (Retinoid X receptor-alpha)
		ERRα (Estrogen-related receptor-alpha)
		GCNF (Germ cell nuclear factor)
		TR2 (Testicular receptor)
	IIC	PPAR γ (Peroxisome proliferator-activated receptor-gamma)
		LXRα (Liver X receptor-alpha)
		GR (Glucocorticoid receptor)

**FIGURE 1:**
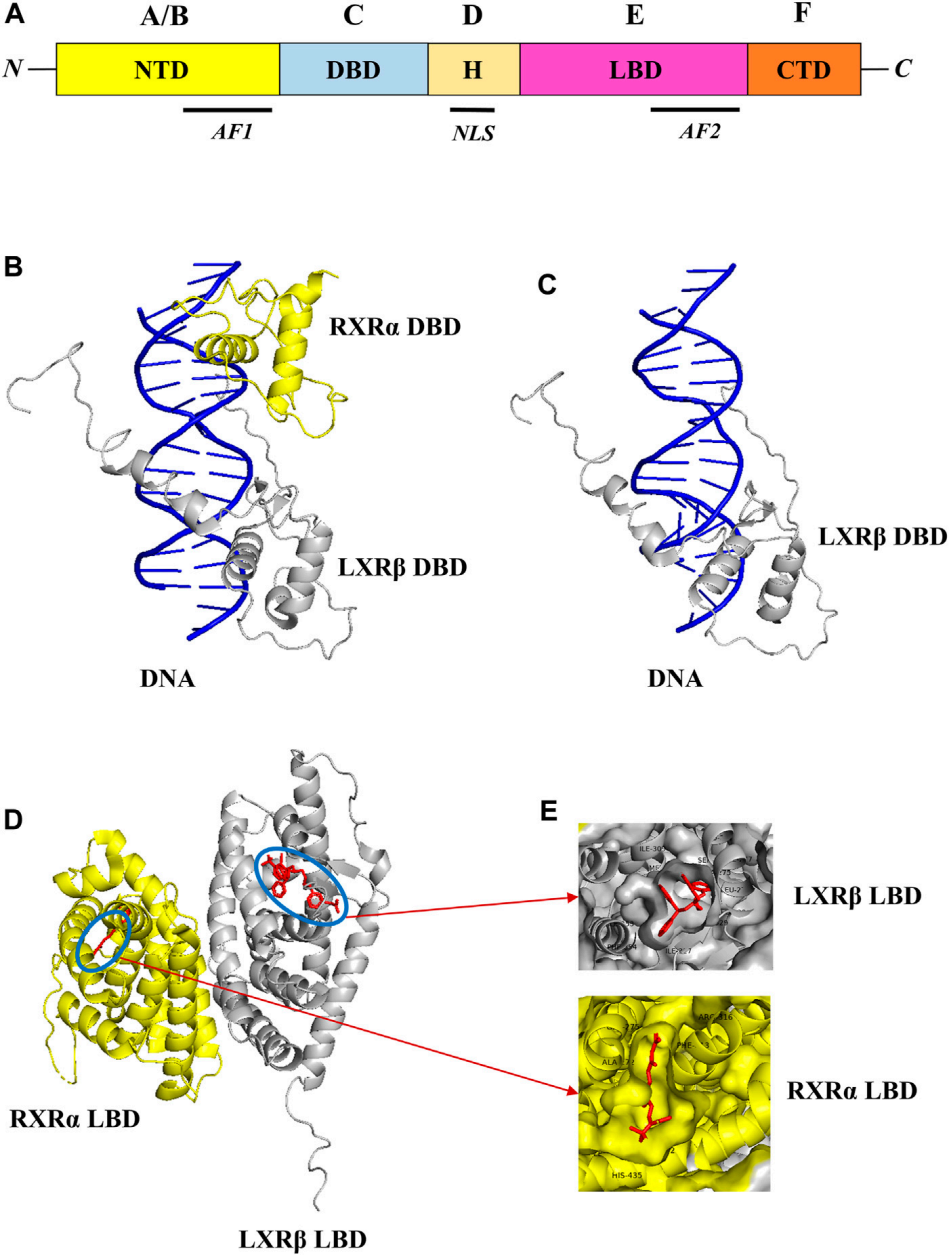
Structural domain organization of LXR. LXRs generally consist of N-terminal domain (NTD), DNA-binding domain (DBD), Hinge region **(H)**, Ligand binding domain (LBD), and C-terminal domain (CTD) **(A)**. The RXR-α (yellow color)/LXR-β (grey color) heterodimer with DNA complex (PDB ID: 4NQA) was retrieved from the PDB database and visualized through Pymol. The DBD of RXR-α/LXR-β heterodimer with DNA complex was shown in **(B)**, and the DBD of LXR-β with DNA complex was shown in **(C)** respectively. Similarly, the LBD of RXR-α/LXR-β heterodimer with ligands was shown in **(D)**, and ligand binding pattern in RXR-α LXR-β and also amino acids around 4 Å was shown in **(E)** respectively.

Liver X receptors (LXRs) are a type of nuclear receptor that is involved in glucose and lipid metabolism, cholesterol transport and metabolism, and also plays a significant role in modulating inflammatory responses (Schulman, 2017). LXRs are classified into two subtypes, namely, LXRα and LXRβ that are encoded by genes NR1H3 and NR1H2, and located in chromosome 11p11.2 and chromosome 19q13.3 respectively (Apfel et al., 1994; Shinar et al., 1994; Willy et al., 1995). The structural domain organization of LXRs consists of the N-terminal domain (NTD), DNA-binding domain (DBD), Hinge region (H), Ligand binding domain (LBD), and C-terminal domain (CTD) respectively as shown in Figure 1 (Rigsby and Parker, 2016; Burley et al., 2023). The NTD is a variable domain that consists of transactivation factor 1 (AF1) in which other proteins could bind and initiate the transcriptional activities; the DBD domain is conserved and consists of two zinc finger DNA motifs; the hinge region has the nuclear localization sequence (NLS) which helps in the translocation of LXRs into the nucleus from the cytoplasm; LBD is also conserved and consists of transactivation factor 2 (AF2) which in the dimerization and also has a CTD domain which is short and variable. Generally, LXRs heterodimers with RXRs, as the LXRα isoform binds with RXRβ and LXRβ isoform binds with RXRα respectively (Folkertsma et al., 2004; 2005; Fitz et al., 2019).

LXRs are associated with various diseases such as cardiovascular diseases (Tontonoz and Mangelsdorf, 2003), Parkinson’s disease (Alnaaim et al., 2023), Atherosclerosis (Endo-Umeda et al., 2022), Huntington’s disease (Courtney and Landreth, 2016), and several cancers (Carpenter et al., 2019; Piccinin et al., 2021). LXRs can regulate the cell cycle in various cancers by inducing CDK inhibitors like p21, inhibiting S-phase Kinase-associated protein (SPK2), and reducing the transition of androgen-dependent to androgen-independent cancers (Piccinin et al., 2021). LXRs have emerged as a potential therapeutic target in various cancers which regulates their tumor growth. LXRs agonists were reported to reduce the cellular proliferation, invasion, and metastasis of melanoma, oral and breast carcinoma (Pencheva et al., 2014; Hu et al., 2017a). LXRs were also known to play a significant role in immune responses, and thus it has greater potential when combined with immunotherapeutic agents in cancer immunotherapy (Tavazoie et al., 2018). Though metabolites such as fatty acids and oxysterols are commonly involved in LXRs regulation and cancer metabolism, these two events could be studied together with respect to understanding the role of LXRs ligands in the altered tumor microenvironment (TME) (Zhao et al., 2019).

## 2 LXRs in cancer

### 2.1 Lung cancer

Lung cancer is the second most lethal and most frequent malignant neoplasm occurring worldwide which is classified into small cell lung carcinoma (SCLC) and non-small cell lung carcinoma (NSCLC) (Sung et al., 2021). LXR activation is associated with reduced cellular proliferation, metastasis, and angiogenesis of lung cancer cells. Due to the EGFR mutations in NSCLC, it is becoming resistant to Epidermal growth factor receptor tyrosine kinase inhibitors (EGFR-TKIs), and recent studies indicated that the LXR activation inhibits lung cancer proliferation and metastasis and also shows synergistic effect when combined with EGFR-TKIs (Liu et al., 2018). In a study, the T0901317 was reported to exhibit a synergistic effect to the gefitinib (EGFR-TKI) in EGFR-TKI-resistant A549 cells and inhibited proliferation and metastasis by Protein Kinase B (AKT) inactivation. In combination with gefitinib, it inhibits invasion and migration confirmed in both *in vitro* and *in vivo* studies, however, T0901317 does not have any effect when used alone (Gao et al., 2016). Some reports also highlighted that the LXRβ activation by T0901317 suppressed the invasion and metastasis, reduced the Matrix Metalloproteinase-9 (MMP-9) levels which promote metastasis by extracellular matrix degradation, and inhibited the MAPK and NF-κB/MMP-9 signalling pathways in A549 cells *in vitro* and BALB/c nude mice *in vivo* (Chen et al., 2017; Lou et al., 2019).

The combination of efatutazone (PPARγ agonist) with T0901317 resulted in the reduced proliferation of lung cancer cells via targeting the PPARγ/LXRα/ABCA1 pathway (Ni et al., 2018). GW3965 was also reported to possess a synergistic effect in overcoming gefitinib resistance against the gefitinib-resistant lung cancer cells by inhibiting Nuclear factor kappa B (NF-κB), and similar to T0901317 it does not have any effect when treated alone (Hu et al., 2017b). In general, the suppression of Myeloid-derived suppressor cells (MDSCs) sensitizes the non-small cell lung carcinoma towards radiotherapy. Notably, GW3965 has the potential to reduce the levels of MDSCs which generally promote metastasis, angiogenesis, and resistance inside the TME (Liang and Shen, 2020). Chemosensitization and radiosensitization of several drugs are being studied to develop potential combination therapies in various cancers. GW3965 depletes the MDSCs in TME and promotes radiosensitization in NSCLC (Liang and Shen, 2020). In macrophages, it promotes radiosensitization by regulating macrophage survival and inflammatory responses (Tabraue et al., 2019). RGX-104 was observed to inhibit cellular proliferation, inducing the apoptotic signals, inhibiting MDSCs, and also sensitizing to radiotherapy with reduced immunosuppressive events (Liang and Shen, 2020). Collectively, it was observed that the combination of LXR agonists with other antineoplastic agents has a potent effect against tumor growth and proliferation, and does not have a significant effect when treated alone (Gangwar et al., 2022).

### 2.2 Breast cancer

Breast cancer (BC) is the most common malignancy in women globally with high incidence and mortality rates and also exhibits resistance to some of the existing therapeutics (Mekala et al., 2018; Sannappa Gowda et al., 2023). LXRs usually bind with RXRs and this heterodimer complex regulates the tumor growth and metabolism, and also this LXR/RXR signalling pathway was reported to play a significant role in the regulation of breast cancer progression (Fitz et al., 2019; Torres-Luquis et al., 2019). From a retrospective German cohort study (*n* = 305), it was identified that LXR localization was usually observed in both nuclei and cytoplasm, and its expression has a positive correlation with the expression of progesterone receptor (PR) and Estrogen receptor (ER). They concluded that the LXR could be used as a poor prognostic marker in breast cancer patients (Shao et al., 2021). Cytochrome P450 (CYP450) enzymes play a crucial role in xenobiotic and drug metabolism, and different isoforms of CYP450 are involved in the metabolism of specific types of drugs/compounds (McDonnell and Dang, 2013). In general, the CYP7B1 expression is reduced in triple-negative breast cancer (TNBC) cells. CYP7B1 usually breaks down the endogenous cholesterol metabolite 27-hydroxycholesterol (27HC) in TNBC cells, and the accumulation of 27HC activates LXR thus further regulating the proliferation of TNBC cells (Nazih and Bard, 2020). Inversely, LXRα/β inhibition was reported to induce tumor elimination in TNBC via enhancement of CD8^+^ T-cell cytotoxicity, mitochondrial metabolism activity, restoration of cytotoxic T-cell clonal expansion and plasma membrane localization (Carpenter et al., 2019). Also, the LXRs activation by inverse agonist SR9243 suppresses the activation of M2 polarization and MDSCs and promotes the migration of dendritic cells (DCs), and also promotes the CD8^+^ T-cell tumor infiltration.

Alternative splicing is an important molecular event in cancer that regulates tumor progression, modulates the functions of intracellular and extracellular components, and is being studied for its therapeutic possibilities in various cancers (Qi et al., 2020). In a study, the truncated LXRs splice variants were observed to be associated with better prognosis in TNBC. They have reported 7 LXRs splice variants, in which 3 LXRs splice variants are newly reported such as LXRα4 (has different AF1 region), LXRα5 (lacks AF1 and LBD regions in greater level), and LXRβ1(has different AF1 region) respectively (Lianto et al., 2021). GW3965 downregulates the transcription factor E2F2 expression, and reduces its attachment to the target genes (mostly cis regions), and also downregulates the genes involved in the proliferation, replication, and cell cycle in BC *in vitro* (Nguyen-Vu et al., 2013). LXR agonist T0901317 inhibited the proliferation and metastasis of butylated hydroxytoluene-induced breast cancer in BALB/c mice *in vivo* and also increased the IFN-γ levels which have a negative correlation with progression (Wang et al., 2016). LXRs are generally activated by the binding of LXR ligands. In another work, researchers reported that phytosterols such as β-sitosterol campesterol have the ability to inhibit the oxysterols (LXR ligands) such as 25-OHC, (25R)26-OHC and 24(S),25-EC thus destructing the LXR activation of oxysterols BC cells *in vitro* (Hutchinson et al., 2019). LXRs were also reported to inhibit the BC proliferation of estrogen-dependent cells by regulating the expression of Estrogen sulfotransferase (EST) and thus play a vital role in estrogen metabolism (Gong et al., 2007; Ju et al., 2017).

### 2.3 Colorectal cancer

Colorectal cancer (CRC) is the second most frequent cancer followed by lung cancer with 10% incidence and 9.4% mortality rates worldwide in 2020 (Dekker et al., 2019; Xi and Xu, 2021). Altered lifestyles such as smoking, alcohol consumption, lack of exercise, and obesity are the major factors of CRC progression. Recently, the lipid metabolism dysregulation mediated by LXRs has been studied to understand the role of LXRs in CRC tumorigenesis (Liu et al., 2019; Lewandowska et al., 2022). In the intestine, cholesterol accumulation is associated with CRC development and it is tightly regulated by LXRs, and even the low expression of LXRα and LXRβ isoforms are negatively correlated with CRC progression confirmed in a cohort study (*n* = 37) (Sharma et al., 2019). LXRs activation in colorectal cancer cells induces apoptosis and inhibits cell proliferation, angiogenesis, and metastasis. Also, they were observed to bind to β-catenin and mediate the expression of β-catenin such as c-myc (Piccinin et al., 2021). T0901317, GW3965 and 22[R]-HC activates the LXRs which further inhibits proliferation, induces apoptosis and cell cycle arrest, and reduces the expression of S-phase–associated kinase protein-2 (Skp2) (Nakayama et al., 2000; Uno et al., 2009). GW3965 induces caspase-dependent apoptosis in Apc^min/+^ mice and AOM-/DSS-Treated Mice *in vivo* and also induces cell cycle arrest at the G1/S phase, inhibits the CRC tumorigenesis in xenograft animal models (Lo Sasso et al., 2013).

The NLS region in the DBD of LXR mediates its localization into the nucleus (Fitz et al., 2019). In a study, it was reported that, unlike LXRα, the LXRβ isoform localizes into the nucleus of CRC and is able to induce pyroptosis (Courtaut et al., 2015). They also observed that the LXRβ activation by T0901317 is strongly associated with the truncated RXRα and inhibits cell proliferation. In another study, the LXRβ was reported to interact with Pannexin-1 (Panx1) which usually activates the P2X7 receptors (P2X7R) and triggers cell death, that induced pyroptosis in caspase-1-dependent manner in CRC (Derangère et al., 2014; Loureiro et al., 2022). While the LXRα expression levels were observed to be lower in CRC than the normal cells, their activation via suitable ligands triggers the caspase-mediated cell death, inhibits the cell cycle, and induces apoptosis (Han et al., 2023). A recent study investigated the SR9243-loaded immunoliposomes effect on CRC and found that LXR activation was mediated by SR9243, and immunoliposomes showed cytotoxic effect specifically in CD133+ cancer stem cells, and they also suggested the dual targeting is also an alternative and potent strategy (Dianat-Moghadam et al., 2023). It also inhibits the proliferation, migration, and clonogenic of human colorectal CD133 cells in a dose-dependent manner. Calreticulin translocation triggers the phagocytic responses, and the high mobility group box 1 protein (HMGB1) is released by ferroptosis-induced cells. Notably, T0901317 was reported to induce calreticulin translocation and HMGB1 levels (Lu et al., 2015; Wu and Yang, 2018). Collectively in colorectal cancer, the LXRs activation blocks the Wnt pathway by binding to β-catenin, and significantly inhibits the expression of its transcriptional targets such as c-myc; induces pyroptosis and apoptosis via caspase-dependent mechanism, inhibits proliferation, cell cycle, angiogenesis, and migration.

### 2.4 Liver cancer

Hepatocellular Carcinoma (HCC) is one of the deadliest cancers and ranked sixth among other cancers with 4.7% incidence and 8.3% mortality rates worldwide in 2020 (Sung et al., 2021; Banini et al., 2022). Hepatitis B Virus, Hepatitis C Virus, cirrhosis, MAFLD, and metabolic dysfunction-associated steatohepatitis (MASH) are the main factors for the chronic conditions of liver diseases followed by the alterations and modifications of cellular components (Karunakara et al., 2022; Suresh et al., 2023). Some studies reported that in viruses-induced HCC, the HCV core protein, non-structural protein 5A, and HBV X protein were degraded which was mediated by sterol-regulatory-element-binding protein overexpression in LXRα/RXRα-dependent pathway (Moriishi et al., 2007; Lima-Cabello et al., 2011). LXRs regulate the cholesterol metabolism in HCC via targeting various signalling pathways such as IL-6/JAK/STAT3 signalling and mediating the transforming growth factor β activity (Bellomo et al., 2018; Xie et al., 2022). Activated LXRα inhibits the Snail protein expression in the TGFβ-dependent pathway due to LXRα and TGFβ crosstalk in HCC and induces intracellular ROS levels and inhibits proliferation (Bellomo et al., 2018). In contrast, T0901317 activated LXRα inhibits alpha-smooth muscle actin or α-SMA (ACTA2) and negatively regulates the TGFβ differentiation in HCC resulting in the increase in the fatty acid synthase (FASN) expression levels the inhibition of tumour growth (Morén et al., 2019). A study proposed that the LXRs are potent prognostic biomarker in HCC patients, which inhibits the expression levels of MMP-2 and MMP-9 via NFκB pathway upon activated by agonist GW3965 (Long et al., 2018).

Forkhead box protein M1 FOXM1 are transcription factors that regulate the expression of genes such as Cdc25A, cyclins, and p21 which regulate the cell cycle and promote the HCC progression, thus making FOXM1 a potential target of HCC. A study in 2014, reported that the LXRα activation by GW3965 and TO901317 downregulates the expression of FOXM1 and induces cell cycle arrest in HCC cells (Hu et al., 2014). A novel mechanism of LXR-activated tumor suppression via the lncRNA/miRNA/mRNA axis of HCC cells was reported by (He et al., 2020). They proved that the activated LXRα downregulates the expression of lncRNA highly upregulated in liver cancer (HULC), which further upregulates HULC miRNA target miR-134-5p expression levels. Thus the FOXM1 mRNA levels were inhibited by the upregulation of miR-134-5p levels in HCC cells via the HULC/miR-134-5p/FOXM1 axis. A recent study investigated the association between LXRs and RALBP1 in HCC progression, in which they found that TO901317 activated LXRα upregulation of the expression levels of RALBP1 associated Eps Domain Containing 2 (REPS2) which shatters the AKT/NF-κB and MAPK pathways by EGFR inhibition in HCC cells (He et al., 2023).

Phytoconstituents such as alkaloids, flavonoids, and other polyphenols are heavily reported for their versatile pharmacological activities and potent anticancer potential against various cancers such as lung, breast, pancreatic, colon, and liver cancer. Captivatingly, a natural steroidal lactone Withaferin A isolated from Withania somnifera acts as a LXRα agonist, and inhibits the expression of various transcriptional targets such as serpin F1, Angiogenin, Endothelin-1, PAI-1, and ICAM-1 in HCC and even inhibits its proliferation, angiogenesis and migration (Shiragannavar et al., 2020; Shiragannavar et al., 2022; Shiragannavar et al., 2023). Similarly, Bergapten (natural coumarin) was also reported to act as the LXRα/β agonist and inhibits the HCC progression via regulating the PI3K/AKT and IDOL/LDLR signalling pathways. Also, it downregulates SREBP1 and FASN levels via AKT inhibition and upregulates ABCA1 levels in HCC (Pattanayak et al., 2018). Collectively in HCC, mostly activated LXRα (LXRβ was not much reported) plays a crucial role by regulating the transcriptional targets, inhibiting the cell cycle regulators, epigenetic regulation of lncRNA/miRNA/mRNA axis, and inducing apoptosis and inhibiting cell proliferation, differentiation and migration.

### 2.5 Pancreatic cancer

Pancreatic ductal adenocarcinoma (PDAC) is a lethal form of pancreatic cancer that has a poor prognosis and ranked seventh among other cancers with 2.6% incidence and 4.7% mortality rates worldwide in 2020 (Sung et al., 2021). LXRs activation was reported to inhibit the PDAC progression via apoptosis induction, cell cycle regulators inhibition, and inhibition of proliferation and metastasis (Ramalingam et al., 2023b). Fatty acid metabolism is highly associated with pancreatic cancer tumorigenesis and some studies reported that LXRs inhibit the transcriptional targets FASN, and SREBP1C that are involved in fatty acid metabolism thereby inhibiting cancer growth (Joseph et al., 2002). A recent study by Widmann and others reported that the LXR agnostic GAC0003A4 induces apoptosis and necroptosis via cell death pathways, and inhibits the proliferation of BxPC-3, MIA PaCa-2, and PANC-1 pancreatic ductal adenocarcinoma cells by disruption of the metabolism of cholesterol and ceramide in a concentration-dependent manner (Widmann et al., 2023). Similarly, the LXR agonists GW3965 and T0901317 inhibited the proliferation of BxPC-3, MIA-PaCa-2, and PANC-1 pancreatic ductal adenocarcinoma cells mainly by downregulating the expression of EGFR and its transcriptional target SKP2, however, LXR knockdown promoted the pancreatic cancer progression (Candelaria et al., 2014). In a previous study, GW3965 induced LXR activation was observed to induce cell cycle arrest at the G1 phase and inhibit the key genes involved in PDAC proliferation, growth, and metastasis (Candelaria et al., 2013). While, the LXR inverse agonist GAC0001E5 was reported to disrupt the glutamine metabolism which produces glutamate to synthesize Glutathione-S-transferase. And also observed to increase the intracellular ROS levels which triggers the apoptotic mediated cell death of PDAC cells. They also reported that GAC0001E5 downregulates the expression of genes such as Glutamate oxaloacetate transaminase 2 involved in glutamine metabolism in PDAC (Srivastava et al., 2020). In contrast, other LXR agonists such as GW3965 and T0901317 do not induce apoptosis in PDAC cells (Candelaria et al., 2014).

Oncogenic KRAS induces tumorigenesis in PDAC and also alters the downstream effectors and PDAC hallmarks. In KRAS-mutated PDAC cells, the LXR inverse agonists GAC0001E5 and GAC0003A4 were known to inhibit cell proliferation and growth by targeting the signalling pathways involved in KRAS mutation (Karaboga et al., 2020). Though the cholesterol metabolism was known to be altered in cancer cells due to LXR activation, they can even regulate the oxysterol metabolism. Unlike normal cells, in LXR activated PDAC cells it inhibits the expression of ATP-binding cassette transporter (ABCA1), ATP-binding cassette sub-family G member 1 (ABCG1) and ATP-binding cassette sub-family G member 5/8 heterodimer (ABCG5/8), promotes the expression of Niemann-Pick C1-Like 1 (NPC1L1), Sulfotransferase family cytosolic 2B member 1 (SULT2B1b) and Multidrug resistance-associated protein 1 (ABCC1), and also inhibits the CYPP450 enzymes like CYP2A1/4A1 respectively (Moschetta, 2011; Bovenga et al., 2015). LXR agonists have the potential to significantly regulate various pathways involved in PDAC progression and tumorigenesis. GW3965 inhibits the expression of genes involved in pancreatic cancer proliferation such as proliferating cell nuclear antigen (PCNA), downregulated p-S6K1 and induced apoptosis, and upregulates ATF4 and TXNIP. And also observed to induce cell cycle arrest via targeting the ATF4/TXNIP/REDD1/mTOR axis irrespective of AMPK activation in PDAC cells (Chen et al., 2022). The sterol response element binding factor-1 (SREBF1) was known to be involved in the DNA repair mechanisms in cancer cells, the activated LXRs are reported to inhibit the activity of SREBF1 via LXR-SREBF1-PNKP axis and significantly promote apoptotic mediated cell death of pancreatic cancer cells (Yang et al., 2020; Han et al., 2023). The squalene epoxidase (SQLE) promotes pancreatic cancer progression via Src/PI3K/Akt signalling pathway, and LXR agonist was known to inhibit the Src/PI3K/Akt axis, and thus they suggested the combination of SQLE and LXR agonists could possibly shut down PDAC progression by inhibiting the high cholesterol uptake in the tumor microenvironment (Xu et al., 2023).

### 2.6 Blood cancer

Hematological cancers such as acute myeloid leukemia (AML), chronic lymphocytic leukemia (CLL), acute lymphoblastic leukemia (ALL) [2.5% incidence and 3.1% mortality rates in 2020], and Multiple myeloma (MM) [0.9% incidence and 1.2% mortality rates in 2020] are the common types worldwide (Sung et al., 2021). The LXR activation in hematological cancers plays a vital role in proliferation, lipid and cholesterol metabolism, metastasis, and tumor immunity (Komati et al., 2017). In multiple myeloma cells, the LXRs activation by GW3965 hydrochloride and LXR-623 agonists enhanced the NK cell-cytotoxicity activity by upregulating the NKG2D ligands such as MHC class I polypeptide-related sequence-A (MICA) and MHC class I polypeptide-related sequence-B (MICB) respectively. LXR activation alters the intracellular cholesterol levels, promotes ABCA1 expression levels, promotes cholesterol efflux, promotes apoptosis, and inhibits the degradation and recycling of cholesterol by MICB (Bilotta et al., 2019). In Jurkat and SupT1 ALL cells, the GW3965 was reported to upregulate the Suppressor of cytokine signaling 3 (SOCS3) levels, promote apoptosis, and inhibit proliferation and colony formation. Also, GW3965 did not regulate the expression of E2F family members such as E2F1, E2F2, and E2F3a, and thus they proposed that SOCS3 as a potential target for leukemia (Zhang et al., 2016).

Geyeregger R et al., reported that the LXR agonists T0913017 and GW3965 inhibit the phosphorylation of cyclin B, cyclin D3, cyclin E; inhibit the IL-2-induced proliferation and induce cell cycle arrest at G1/S phase and G2/M phase; promotes apoptosis; inhibits the expression of bcl-2 and MMP-9 genes in Kit225 T-CLL cells, T cell blasts from healthy donors, and primary T and B cells from CLL patients respectively (Geyeregger et al., 2009). Dendrogenin A (DDA), a cholesterol metabolite, a partial agonist of LXR and tumor suppressor was observed to induce LXRβ-dependent lethal autophagy in AML cells confirmed in both *in vitro* and *in vivo* studies by (Segala et al., 2017). They reported that DDA promoted autophagy by upregulating the expression of Nur77, Nor1, and LC3 and downregulating the expression of 3β-hydroxysterol-Δ8,7-isomerase (D8D7I). DDA treatment of AML cells, promoted SCD1, SREBF1, LC3s, and NR4As expression levels and suppressed ABCA1, and LDLR expression levels (Segala et al., 2017). In acute myeloid leukemia and T-acute lymphoblastic leukemia cells, the T0901317 and GW3965 mediate the cholesterol efflux by upregulating the expression of ABCA1 and ABCG1, and downregulating the expression of LDLR and VLDLR levels; promoting apoptosis; inhibited proliferation, inhibited NF-κB activation, inhibits the IL-3–induced signaling pathway thereby by inhibits the phosphorylation of STAT5 and Akt in AML cells (Ceroi et al., 2016). Comparatively, the role of activated LXRs in hematological cancers is less explored and more studies have to be performed to understand the multifaceted role of LXRs in blood cancers.

**FIGURE 2:**
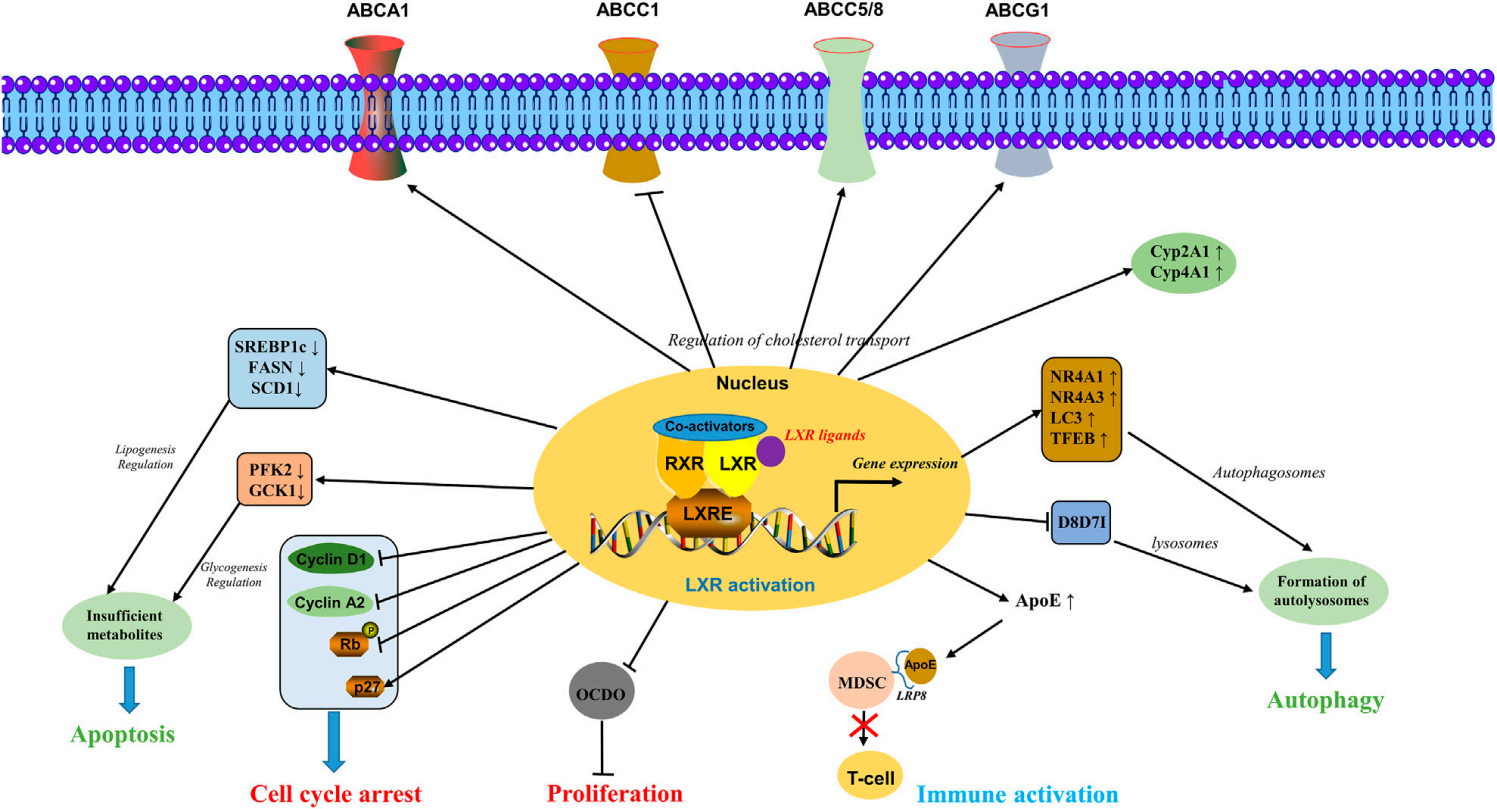
Molecular mechanisms involved in the activation of LXRs. LXRs activation plays a crucial role in cancer including the regulation of cholesterol transport via ABC transporters and Cyp450 enzymes, apoptosis induction by glycogenesis and lipogenesis regulation, autophagy induction via formulation of autolysosomes, cell cycle arrest by regulating key genes involved in cell cycle, immune activation by T-cell via inhibiting the MDSCs, inhibiting cellular proliferation by inhibiting the expression of tumor promoter OCDO.

Overall, the LXRs activation plays a crucial role in cancer including the regulation of cholesterol transport via ABC transporters and Cyp450 enzymes, apoptosis induction by glycogenesis and lipogenesis regulation, autophagy induction via formulation of autolysosomes, cell cycle arrest by regulating key genes involved in cell cycle, immune activation by T-cell via inhibiting MDSCs, inhibiting cellular proliferation by inhibiting the expression of tumor promoter 6-oxo-cholestan-3β,5α-diol (OCDO) (Flaveny et al., 2015; Poirot and Silvente-Poirot, 2018; Tavazoie et al., 2018) as shown in Figure 2.

## 3 LXRs ligands

### 3.1 Synthetic ligands

**FIGURE 3:**
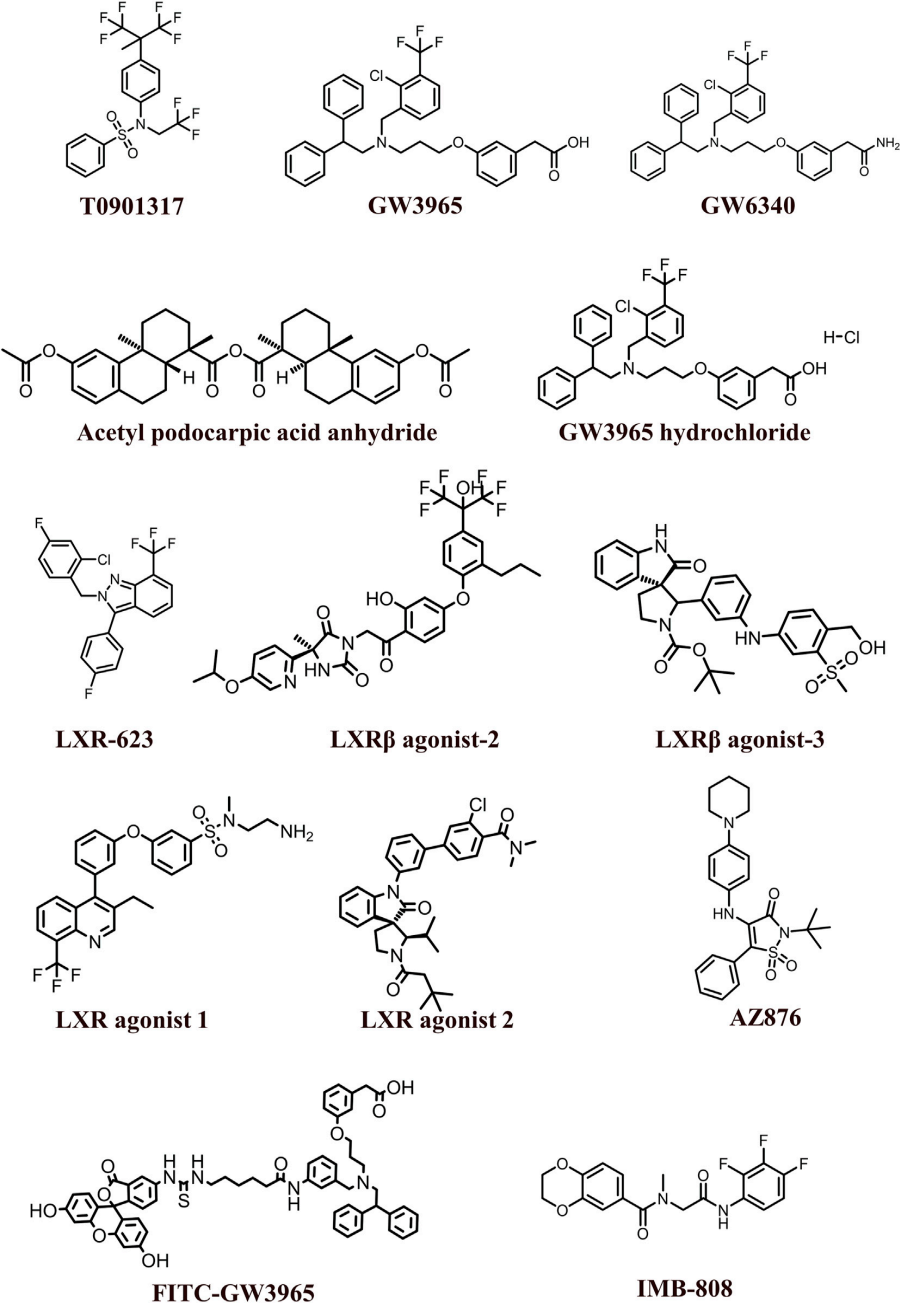
2D structures of the synthetic LXR agonists.

**FIGURE 4:**
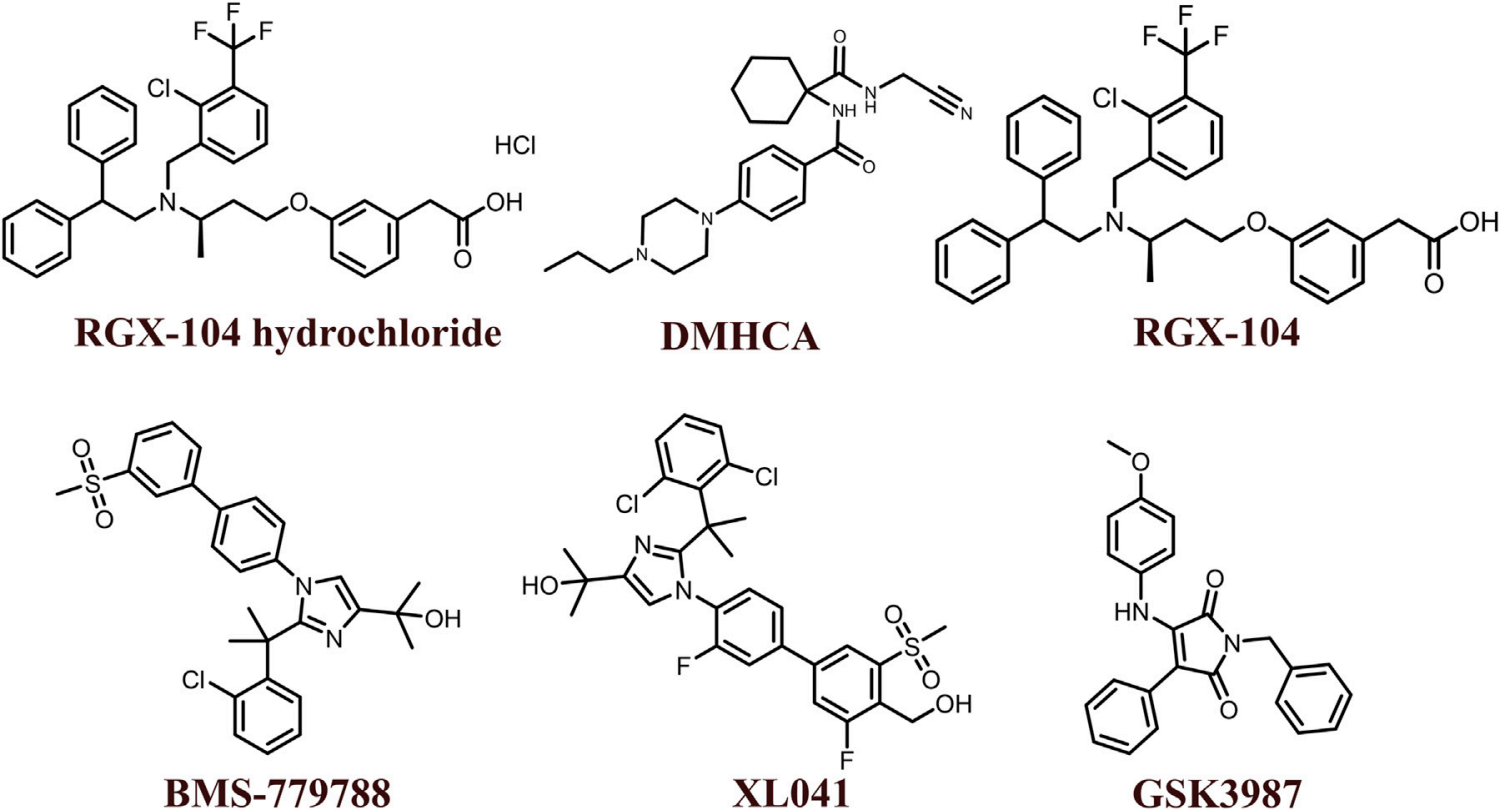
2D structures of the synthetic LXR agonists.

The 2D structures of synthetic LXR agonists such as T0901317, GW3965, GW6340, Acetyl podocarpic acid anhydride, GW3965 hydrochloride, LXR-623, LXRβ agonist-2, LXRβ agonist-3, LXR agonist 1, LXR agonist 2, AZ876, FITC-GW3965, IMB-808, RGX-104 hydrochloride, DMHCA, RGX-104, BMS-779788, XL041, GSK3987 were provided in Figures 3, 4 and other details were elaborated in Table 2. Although there are several synthetic LXR agonists were identified, T0901317, GW3965, GW6340, LXR-623, and RGX-104 were well-known LXR agonists which were discussed more in detail.

**TABLE 2:** Physiochemical properties, pharmacokinetics, pharmacodynamics and molecular mechanisms of LXRs ligands.

Agonists/Antagonists	Molecular formula	Molecular weight (g/mol)	Reversible/irreversible	Selectivity	Bioavailability	IC_50_	Disease type	Cell line/animal model	Mechanism/outcomes	References
T0901317	C_17_H_12_F_9_NO_3_S	481.33	Reversible	Highly selective	Orally active	EC_50_ = 20 nM	HCC	HepG2	CYP7A1↑, SCD-1↑, SREBP-1↑, SQS↓,	Schultz et al. (2000)
									HMG CoA S↓	
GW3965 hydrochloride	C_33_H_32_Cl_2_F_3_NO_3_	618.51	Reversible	Selective	Orally active	0.7 nM	LC	HCC827/GR	NF-κB↓, p-AKT↓, Apoptosis↑,	Hu et al. (2017a)
									Sensitizes gefitinib in EGFR mutant cells	
27-Hydroxycholesterol	C_27_H_46_O_2_	402.65	Reversible	Selective	-	Ki = 1.32 μM	HCC, BC	HepG2, MCF7	ERBB4↓,	DuSell et al. (2008)
									IL1-R1↓, SMAD3↓,	
									Regulates ERα transcriptional activity at pS2 promoter	
LXR-623	C_21_H_12_ClF_5_N_2_	422.78	-	Selective	Orally active	LXRα = 24 nM, LXRβ = 179 nM	GBM	U87MG, U87EGFRvIII iRFP720 and GBM39 IRFP720 xenograft mice	ABCA1↑,	Villa et al. (2016)
									ABCG1↑,	
									Ido↑,	
									Apoptosis↑, cholesterol level↓	
GW6340	C_33_H_32_ClF_3_N_2_O_2_	581.07	Reversible	Selective	Intestine specific	1.78 μM	HCC	HepG2	ABCA1↑,	Li et al. (2017)
									Apoptosis↑, promotes cholesterol efflux	
22(R)-Hydroxycholesterol	C_27_H_46_O_2_	402.65	Reversible	Selective	-	EC_50_ = 325 nM	HCC, BC, PC	HepG2, MCF7, LnCap	Skp2↓, cyclin A2↓, cyclin D1↓, p53↑, Cell cycle arrest at G1 phase	Chuu and Lin (2010)
DMHCA	C_26_H_43_NO_2_	401.63	-	Selective	Oral (Poor)	0.7 nM	BC	MMTV-NeuT/ATTAC mice	CD4^+^ T cells↑, CD8^+^ T cells↑,	Sheng et al. (2021)
									Spp1↓, S100a9↓, Anxa1↓, Mfge8↓,	
									Cd14↓	
AZ876	C_24_H_29_N_3_O_3_S	439.57	Irreversible	Selective	Orally active	7 nM	CH	C57Bl6/J mice	TGFβ↑,	Cannon et al. (2015)
									Angiotensin II↑,	
									SMA↑	
RGX-104	C_34_H_33_ClF_3_NO_3_	596.08	Reversible	Selective	Orally active	LXRα EC_50_ = 84 nM, LXRβ EC_50_ = 20 nM	Cancer patients	TIICs, MDSCs	Tumor growth↓,	Tavazoie et al. (2018)
									MDSCs levels↓,	
									MHC-II levels↑,	
									ApoE↑	
XL041 (BMS-852927)	C_29_H_28_Cl_2_F_2_N_2_O_4_S	609.51	Irreversible	LXRβ Selective	Orally active	EC_50_ = 9 nM	hypercholesterolemic	C57BL/6 mice	SREBP1c↓, FAS↓,	Kirchgessner et al. (2016)
									SCD1↓, cholesterol efflux↑, IL-23α↑	
GSK3987	C_24_H_20_N_2_O_3_	384.43	-	Selective	Orally active	LXRα EC_50_ = 50 nM, LXRβ EC_50_ = 40 nM	HTS	NA	ABCA1↑	Jaye et al. (2005)
BMS-779788	C_28_H_29_ClN_2_O_3_S	509.06	Reversible	Selective	-	LXRα = 68 nM, LXR*β* = 14 nM	atherosclerosis	cynomolgus monkey	ABCA1↑, ABCG1↑,	Kirchgessner et al. (2015)
									Phospholipid levels↓	
RGX-104 hydrochloride	C_34_H_34_C_l2_F_3_NO_3_	632.54	Reversible	Selective	Orally active	-	Cancer patients	TIICs, MDSCs	Tumor growth↓,	Tavazoie et al. (2018)
									MDSCs levels↓,	
									MHC-II levels↑,	
									ApoE↑	
24(S)-Hydroxycholesterol	C_27_H_46_O_2_	402.65	Reversible	Selective	-	130 nM	NB	SH-SY5Y	CaMKII↑,	Kimura et al. (2018)
									RIPK1↑,	
									Necroptosis↑	
IMB-808	C_18_H_15_F_3_N_2_O_4_	380.32	Reversible	Selective	Orally active	LXRα EC_50_ = 0.53 μM, LXRβ EC_50_ = 0.15 μM	Atherosclerosis	RAW264.7, THP-1	Cholesterol efflux↑, lipid accumulation↑	Li et al. (2017)
Acetyl podocarpic acid anhydride	C_38_H_46_O_7_	614.77	-	-	-	0.1 µM	Atherosclerosis	THP-1	Cholesterol efflux↑, phospholipid↑,	Sparrow et al. (2002)
									ABCA1↑,	
									ABCG1↑,	
									SREBP-1c↑	
Nagilactone B	C_19_H_24_O_7_	364.39	Reversible	Selective	-	1 mM	Atherosclerosis	RAW264.7, THP-1, apoE-deficient mice	ABCA1↑,	Gui et al. (2016)
									ABCG1↑,	
									Plasma lipids↑,	
									ABCG5↑,	
									ABCG8↑	
FITC-GW3965	C_59_H_56_N_4_O_9_S	997.16	Reversible	Selective	Orally active	EC_50_ = 190 nM	FBFS	-	Boc group of LBD stabilized the His-Trp activation	Zhang et al. (2019)
GW3965	C_33_H_31_ClF_3_NO_3_	582.05	Reversible	Selective	Orally active	LXRα EC_50_ = 190 nM, LXRβ EC_50_ = 30 nM	Diabetics	Sprague–Dawley rats	PREG↑, PROG↑, DHEA↑,	Mitro et al. (2012)
									DHT↑,	
									17α-E↑	
LXRβ agonist-2	C_32_H_31_F_6_N_3_O_7_	683.59	Reversible	LXRβ Selective	Orally active	LXRα EC_50_ = 200 nM, LXRβ EC_50_ = 0.05 nM	Atherosclerosis	LXRs/GAL4 fused vector in CHO K-1 cells	ABCA1↑,	Koura et al. (2016)
									SREBP-1c↑	
Iristectorigenin B	C_17_H_14_O_7_	330.29	Reversible	Selective	-	18.66 µM	Atherosclerosis	RAW 264.7	SREBP-1c↑,	Jun et al. (2012)
									FAS↑,	
									SCD↑	
LXR agonist 2	C_35_H_40_ClN_3_O_3_	586.16	Reversible	Selective	Orally active	19.7 µM	Atherosclerosis	3T3-L1	SREBP-1c↓, ACC↓,	Chen et al. (2020b)
									FAS↓,	
									SCD-1↓	
LXR agonist 1	C_27_H_26_F_3_N_3_O_3_S	529.57	Reversible	Partially selective	Orally active	LXRα = 1.5 nM,	Atherosclerosis	THP-1	ADCs overcomes the LXR agonists limitations	Lim et al. (2015)
						LXRβ = 12 nM				
LXRβ agonist-3	C_30_H_33_N_3_O_6_S	563.66	Reversible	Selective	Orally active	LXRα EC_50_ = 0.27 μM,LXRβ EC_50_ = 0.09 μM	GBM	U87EGFRvIII, U251	ABCA1↑, IDOL↑, ABCG1↑, APOE↑,	Chen et al. (2020a)
									SREBP-1c↑	
Saikosaponin A	C_42_H_68_O_13_	780.98	Reversible	Selective	Oral	1.7 μM	Osteoarthritis	Primary human osteoarthritis chondrocytes	IL-1β↑,	Gao et al. (2017)
									NF-κB↓	
24-Hydroxycholesterol	C_27_H_46_O_2_	402.65	Reversible	Selective	-	130 nM	NETs	RIP1-Tag2 × RIP1-SULT2B1b double transgenic mice	Cyp11a1↓, Cyp7b1↓,	Soncini et al. (2016)

Note: CH, cardiac hypertrophy; PC, prostate cancer; TIICs, Tumor infiltrating immune cells; MDSCs, Myeloid derived suppressor cells; HTS, High throughput screening; FBFS, fluorescence based fragment screening; NETs, pancreatic neuroendocrine tumor.

T0901317 exhibited synergistic in combination with vitamin D3 by increased expression of ABCA1 levels and reduced expression of cholesterol levels, and significantly promoted apoptosis by upregulating BAX and downregulating Bcl-2 levels in MCF-7 cells and thus observed to prevent hyperlipidemia-mediated estrogen receptor-positive (ER+) BC progression (Munir et al., 2020). When combined with gefitinib, T0901317 reduced the migration and invasion levels of lung cancer via inhibition of the MAPK pathway (Lou et al., 2019), and sensitized the HCC cells towards sorafenib by inhibiting Mesenchymal-Epithelial Transition (MET) and EGFR (Shao et al., 2020). However, even alone it possesses significant anticancer properties, for instance in HCC, T0901317 was reported to upregulate the CYP7A1, SCD-1, and SREBP-1 levels and downregulate the SQS, and HMG CoA S levels (Schultz et al., 2000).

Epithelial-mesenchymal transition (EMT) plays a major role in drug resistance of various cancer types. GW3965 reduced the gefitinib resistance in NSCLC cells by reducing the expression of vimentin which plays a crucial role in EMT (Hu et al., 2017b). In another study, it sensitizes NSCLC cells to gefitinib by downregulating NF-κB and promoting apoptosis (Hu et al., 2017a). Also, when combined with gemcitabine GW3965 inhibited the expression of G1-S phase regulatory protein and promoted apoptosis in a dosage-dependent manner in KRAS mutated BxPC-3, MIA-PaCa-2, and PANC-1 pancreatic ductal adenocarcinoma cells (Candelaria et al., 2014). This observation also indicates that these LXR agonists need to be studied along with mutant KRAS inhibitors such as sotorasib and adagrasib, which could possibly reduce the resistance of KRAS mutant inhibitors and sensitize the PDAC cells. In glioblastoma, GW3965 inhibits EGFR/AKT/SREBP-1/LDLR-dependent pathway and sensitizes the glioblastoma cells towards lapatinib treatment (Guo et al., 2011).

LXR-623 was able to penetrate the blood-brain barrier and was reported to possess significant tumor reduction in glioblastoma cells in a cholesterol-dependent manner (Villa et al., 2016). lncRNAs generally act as competing endogenous RNA and regulate the expression of various mRNAs with sponging miRNAs in various physiological and pathological conditions. LXR-623 was also reported to induce the expression of LINC01125 (sponge miR-19b-3p) which further suppresses the PTEN/AKT/p53 signaling pathway in BC (Wan et al., 2019). Both LXR-623 and GW3965 were reported to have synergistic effects towards BH3 mimetics by downregulating the Bcl-2 expression levels in various cancers such as breast, colon, lung, and glioblastoma confirmed in both *in vitro* and *in vivo* studies (Nguyen et al., 2019b). In combination with gamitrinib, LXR623 synergistically reduced the proliferation of cancer cells by promoting apoptosis via upregulating Bax and downregulating Bcl-2, and this combination even inhibited the expression of Tumor Necrosis Factor Receptor-associated Protein 1. The above observation showcases the importance of the need for combined treatment of LXR623 with gamitrinib in solid tumors (Nguyen et al., 2019a).

GW6340 is an intestine-specific LXR agonist that promotes cholesterol efflux, promotes apoptosis, and upregulates the expression of ABCA1 in HCC (Li et al., 2017). In mice models, it promotes fecal excretion of macrophage-derived cholesterol without alteration of genes included in cholesterol efflux (Yasuda et al., 2010). Though the LXR agonists exhibit significant synergistic effects in combination with the chemotherapy drugs there is a high need for more combination studies. In 2020, a phase 1b study of RGX-104 and docetaxel combination showed their safety and pharmacodynamic profiles in checkpoint inhibitors refractory patients (Lim et al., 2020).

### 3.2 Natural ligands

**FIGURE 5:**
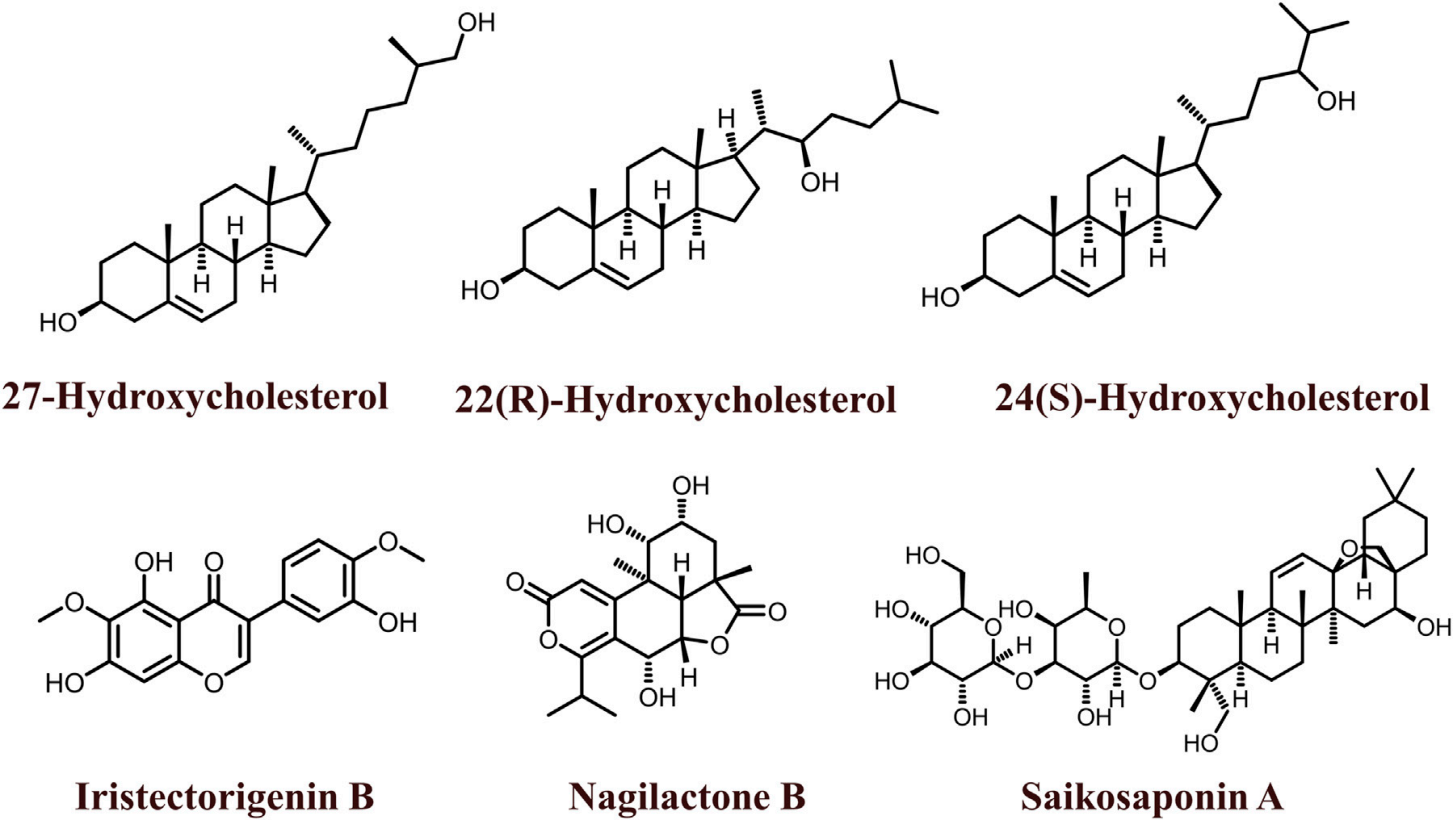
2D structures of natural LXR ligands.

The 2D structures of natural LXR agonists such as 27-Hydroxycholesterol, 22(R)-Hydroxycholesterol, 24(S)-Hydroxycholesterol, Iristectorigenin B, Nagilactone B, Saikosaponin A were provided in Figure 5 and other details were elaborated in Table 2. Although there are several natural LXR agonists were identified, 27-hydroxycholesterol, Withaferin A, and Saikosaponin A were well-known LXR agonists for their significant pharmacological properties as mentioned in Table 2.

27-hydroxycholesterol (27-HA) are partial agonist of LXRs that regulates the expression of genes involved in fatty acid and cholesterol metabolism, and glycogenesis and also plays a major role in cellular proliferation and metastasis in various cancers, especially in breast cancer (Steffensen and Gustafsson, 2004; Vedin et al., 2009). A patient cohort study by Revilla G et al., 2019, demonstrated that the intratumoral accumulation of 27-HA is highly associated with the proliferation and metastasis of papillary thyroid carcinoma patients (Revilla et al., 2019). Epigenetics involves the alteration of epigenetic enzymes, DNA methylation, and histone modifications by HDACs and HATs, even resulting in genomic instability (Sharma et al., 2010). These epigenetic changes often play a crucial role in various cancers such as breast, colon, liver, and pancreas. 27-HA has also been reported to hypermethylate the promoter region of various tumor suppressor genes PTDSS2, DTYN, IDO1, THRA, and MIER in BC (Vini et al., 2022). Conversely, in ER + breast cancer cells, 27-HA promotes proliferation via hypermethylation of CYP7B1 and induction of CCL2, CCL3, and CCL4 chemokine levels (Shi et al., 2019). Additionally, the regulation of the rate-limiting enzyme CYP27A1 of 27-HA is highly associated with the promotion of breast cancer metastasis. Furthermore, it is clearly indicated that 27-HA synthesis is mediated by CYP27A1, binds to ER, acts as a partial agonist towards LXRs, and regulates the expression levels of CYP7B1 by several studies (Abdalkareem Jasim et al., 2022). Another important cohort study reported the negative correlation between circulatory 27-HC levels and CYP27A1, ER, LXRs, and CYP7B1 expression levels in breast cancer (Le Cornet et al., 2020).

Natural compounds such as phytochemical and marine compounds are being extensively studied for their versatile pharmacological activities. Withaferin A isolated from *Withania somnifera* was identified as a novel LXR agonist that inhibits NF-κB, downregulates PEDF, Angiogenin and ICAM-1, induces the expression of ABCA1, ABCG1, and Apolipoprotein (ApoE) levels and thus inhibits the proliferation of HCC cells *in vitro* (Shiragannavar et al., 2020). Withaferin A binding to LXR also inhibits the p50 and p65 protein complexes that form transcriptionally active heterodimers (Shiragannavar et al., 2022). Even at low concentrations, Withaferin A was reported to inhibit the proliferation and migration of Ca9-22 oral cancer cells by inhibiting MMP-2 and MMP-9, upregulating nuclear factor, erythroid 2-like 2 and NAD(P)H quinone dehydrogenase 1 levels, phosphorylating ERK1/2, JNK and p38 levels (Yu et al., 2020). Notably, Withaferin A was also reported to possess dual LXR/FXR receptor activation activity. It has been observed to decrease the levels of hepatic triglycerides, total cholesterol, non-HDL cholesterol, alkaline phosphatase, aspartate aminotransferase, and alanine transaminase, and even inhibits the expression of IL-6 and TNF-α levels confirmed by *in vivo* studies (Sannappa Gowda et al., 2023).

Saikosaponin A isolated from *Bupleurum falcatum* is also an LXR agonist that inhibits the expression of Prostaglandin E2, MMP1, MMP3, and MMP13 levels and phosphorylates NF-κB p65 in human chondrocytes. Alongside it even inhibited the production of nitric oxide representing its activity against osteoarthritis (Gao et al., 2017). Moreover, it also regulates cholesterol transport via activating ABCA1 and ABCG1 and also inhibits cytokines such as TNF-α and IL-6 in macrophages (Wei et al., 2016).

## 4 LXRs in tumor microenvironment and anti-tumor immunity

The tumor microenvironment (TME) is a complex heterogeneity ecosystem of various cells that includes immune cells, stromal cells and other non-cellular components and also plays a major role in tumor promotion, invasion, metastasis, proliferation, and angiogenesis (Arneth, 2019; Baghban et al., 2020; Galli et al., 2020; Lei et al., 2020; de Visser and Joyce, 2023; Liu et al., 2023). In TME, there will be an abundant level of cytokines, chemokines, growth factors, and inflammatory mediators that promote angiogenesis and cancer progression, and multi-drug resistance (MDR) of anticancer drugs (Denisenko et al., 2018; Frisch et al., 2019). The TME is very complex to understand but several research was going to study the complexity of TME for the development of novel therapeutic strategies.

**FIGURE 6:**
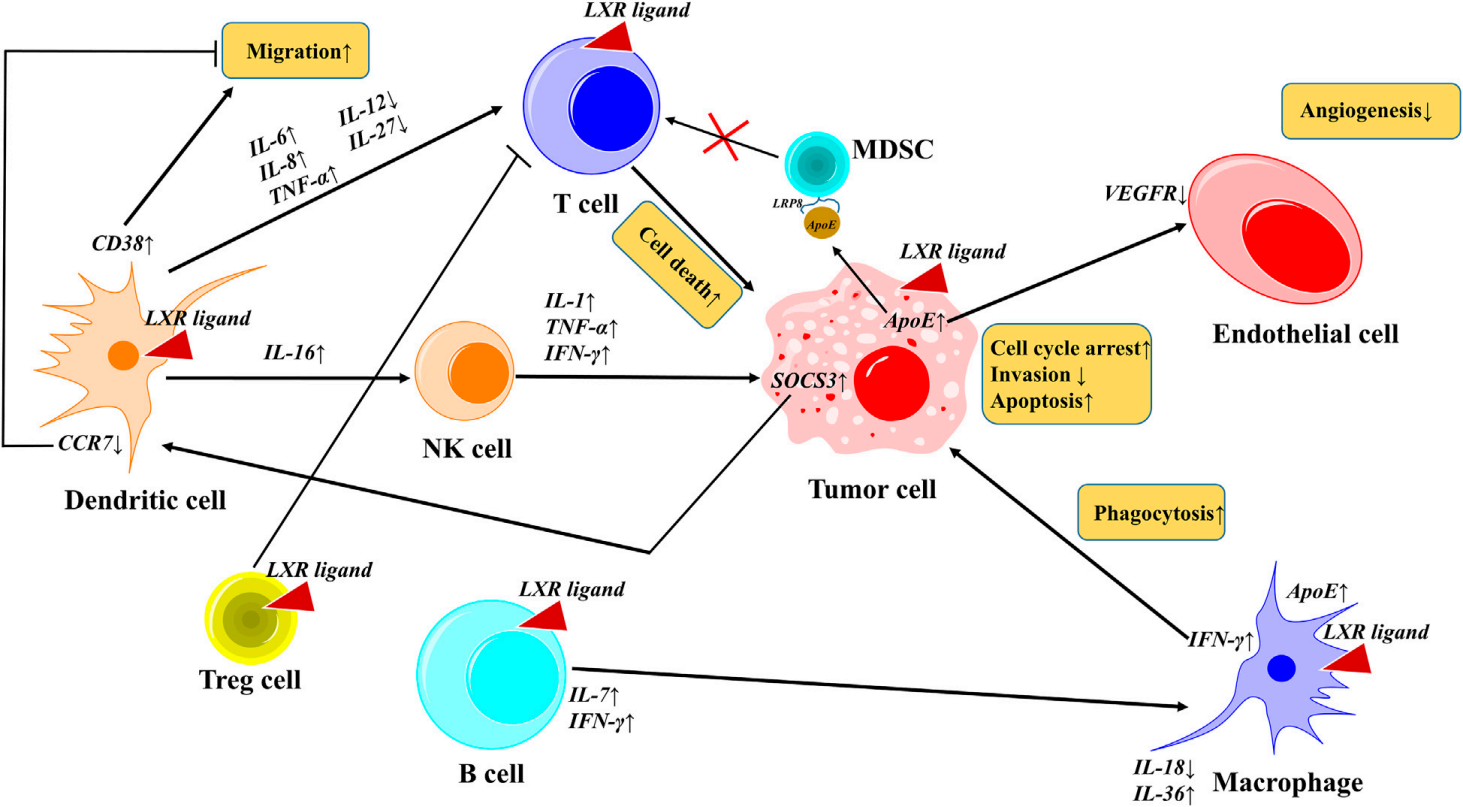
LXRs activation in tumor microenvironment. In TME, activated LXR plays a crucial role in various events such as cell cycle, invasion, migration, angiogenesis, phagocytosis, and cell death by the regulation of various cytokines. Notably, the dendritic cells play a dual role by promoting migration (CD38↑) and inhibiting migration (CCR7↓). These molecular events in the TME mediate the activated-LXR-mediated cancer cell death.

LXRs are reported to play a crucial role in TME such as invasion, migration, proliferation, and tumor promotion, however, their activation by suitable ligands will reverse the mechanism and suppress the tumor progression (Bilotta et al., 2020). Tumor immunity represents the anti-tumor immune response inside the TME, that can regulate the activity of immune cells via the secretion of cytokines and chemokines. In the tumor microenvironment, LXR activation plays a crucial role in various events such as cell cycle, invasion, migration, angiogenesis, phagocytosis, and cell death by the regulation of various cytokines. Also, notably, the dendritic cells play a dual role by promoting migration (CD38↑) and inhibiting migration (CCR7↓). These molecular events in the TME mediate the activated-LXR-mediated cancer cell death (Ju et al., 2017) as shown in Figure 6. LXR activation induces pyroptosis and caspase-1-dependent cell death of CRC (Derangère et al., 2014), and promotes breast cancer via cholesterol metabolism (McDonnell et al., 2014) due to TME heterogeneity (Carpenter et al., 2019). LXR-mediated upregulation of ApoE binds to low-density lipoprotein receptor-related 8 (LRP8) of MDSCs and thus depletes the MDSCs and further promotes T-cell activation (Tavazoie et al., 2018). In a real-time patient setting combination of PD-1 inhibitor and LXR activator promoted the potency of NK cells and CD8^+^ T-cells. CCR7 usually promotes tumor migration in the TME, and notably, the LXR activation inhibits the CCR7 in DC cells by upregulating SOCS3 and thus inhibits cancer migration (Villablanca et al., 2010). However, DC plays a double-edged sword by promoting cancer migration by upregulating CD38 levels (Beceiro et al., 2018).

Reduced cholesterol uptake and its efflux regulation are associated with the decreased cellular proliferation of cancer cells in the TME. Notably, the activated LXRs induce the activation of ABCA1, ABCC5/8 and ABCG1, and inhibit the ABCC1 and thus regulate the cholesterol transport in tumor cells, indicating the activated LXR-mediated cancer cell death in the TME (Tontonoz, 2011). Activated LXRs regulate lipogenesis by inhibiting SREBP1c, FAS, and SCD1, and regulate glycogenesis by inhibiting Phosphofructokinase 2, Glucokinase 1 respectively (Willy et al., 1995; Wang et al., 2018). The NK cell-activating receptor NK group 2-member D (NKG2D)-mediated immune responses play a major role in tumor growth inside the TME. In multiple myeloma cells, LXRs activation enhanced the NK cell-cytotoxicity activity by upregulating the NKG2D ligands, namely, MHC class I polypeptide-related sequence-A (MICA) and MICB respectively (Bilotta et al., 2019). It promotes the expression of MICA by binding to its promoter and degrades MICB. Taken together, the role of activated LXR in TME is both tumor promotion and suppression depending upon the condition such as cholesterol efflux and transport, cytokine and chemokines levels, and further studies are required for a better understanding of LXR activation in TME.

## 5 Precision medicine towards LXR-targeted therapies

Cancer precision medicine has started a significant era for the personalized treatment of individual characteristics of cancer patients, and could also able to predict the cancer risk, early diagnosis, and treatment (combination therapies if needed) among individuals (Hoeben et al., 2021; Lancet, 2021). In conventional cancer treatment strategies, the patients are usually classified only by anatomic site, risk profile, histopathology and cancer stage, which may lead to treatment failure and adverse side effects. Whereas, in cancer precision medicine followed by the conventional classification, the patients will also be grouped individually by their health history, medical record, environmental parameters, and OMICS approaches which include genomics, proteomics, transcriptomics, metabolomics, glycomics, lipidomics, pharmacogenomics and interactomics respectively (Tsimberidou et al., 2020; Edsjö et al., 2023). In the case of LXR-targeted therapies, the LXR ligands/agonists were observed to show two different manners, namely, the same LXR ligand differentially regulates gene expression depending on the tissue/cell type, and different LXR ligands show overlapping but distinct gene expression profiles of the same tissue/cell type which could be harnessed for precision medicine development (Ma et al., 2022). Also, it is evident that the identification of potential biomarkers of activated LXRs in cancer would be helpful in the development of personalized treatment strategies (Ma et al., 2022; Edsjö et al., 2023). For instance, in peripheral blood mononuclear cells (PBMC), monocytes, T-cells and B-cells, activated LXR regulates the expression of ABCA1, ABCG1, and apoE levels in both time and concentration-dependent manner (DiBlasio-Smith et al., 2008). In HLTMs (Human Lung Tissue Macrophages) and THP-1 monocytes. the activated LXR was observed to upregulate the expression of various inflammatory biomarkers such as iNOS, IL-1, CINC-1, and CINC-3; and also upregulates transcription factors such as c-Jun and c-Fos respectively (Birrell et al., 2007). In PBMCs, LXR upregulates the expression levels of cluster of differentiation such as CD82, CD226, and CD244 indicating that it could be activated-LXR mediated biomarkers (Rébé et al., 2012). The above-mentioned biomarkers are associated with the activation of LXR, and further high throughput next-generation sequencing studies would help in the discovery of new potential biomarkers for LXR-targeted therapeutic strategies.

## 6 Conclusion and future perspectives

Cancer has become a serious health burden with constantly increasing incidence and mortality rates every year, mainly due to various molecular alterations inside the cell. Liver X receptors (LXRs) dysregulation is one among them that plays a vital role in cholesterol metabolism, lipid metabolism and inflammation and plays a crucial role in various diseases including cancer. LXRs are differentially expressed in various cancers such as lung, breast, colon, pancreatic, and liver and are known to mediate Wnt, and PI3K/AKT, and MAPK signalling pathways. Activated LXR inhibits cancer cell proliferation, invasion, migration, growth, and progression. LXR regulates variety of molecules involved in cell cycle (Cyclin A2, Cyclin D1, p-Rb, p27); cholesterol efflux (ABCA1, ABCC1, ABCC5/8, ABCG1); Cyp450 enzymes (Cyp2A1; Cyp4A1); apoptosis (Bim, Bax, Bcl-2, Bcl-xL), autophagy (NR4A1, LC3, TFEB), transcription factors (NF-κB) and cytokines (IL-1,6,7,8,16,18,27,36, TNF-α, IFN-γ). Also, LXR modulates cholesterol efflux and transport, lipogenesis, glycogenesis, and angiogenesis and even promotes antitumor immunity in the TME. Altogether, from the above understandings LXRs could be considered as a potential drug target in cancer therapeutics. However, LXRs activation acts as both tumor suppressor and tumor promoter depending upon the TME environment of the cancer type and heterogeneity. Moreover, the upregulation of ABCA1, ABCG1, apoE, iNOS, IL-1, CINC-1, CINC-3, CD82, CD226 and CD244 in LXR activated cells were identified biomarkers. Furthermore, some experimental studies and further considerations are much needed towards LXR-targeted therapies such as,

i. Extensive studies of LXRs role in TME are highly needed for a better understanding of their role and behavior (For instance, LXR-activated dendritic cells either promote migration (via CD38↑) or inhibit migration (via CCR7↓) depending upon the TME condition).
ii. In-depth studies have to be performed to understand the chemo-sensitization and radio-sensitization behaviors of LXR agonists for the development of novel potent cancer therapies.
iii. Combination therapies of LXR agonists along with anticancer drugs are highly needed to improve the synergistic effect among patients.
iv. High throughput next-generation sequencing studies have to be performed to identify more potential biomarkers in LXR-targeted therapies for the development of cancer precision medicine and personalized treatment strategies.

By addressing these gaps, the LXRs could be significantly regarded as potent drug target for the development of novel cancer therapies in the near future.
